# The effect of ‘Traffic-Light’ nutritional labelling in carbonated soft drink purchases in Ecuador

**DOI:** 10.1371/journal.pone.0222866

**Published:** 2019-10-03

**Authors:** Luis A. Sandoval, Carlos E. Carpio, Marcos Sanchez-Plata

**Affiliations:** 1 Department of Agribusiness, Zamorano University, Tegucigalpa, Honduras; 2 Department of Agricultural and Applied Economics, Texas Tech University, Lubbock, TX, United States of America; 3 Department of Animal and Food Science, Texas Tech University, Lubbock, TX, United States of America; Medical University of South Carolina, UNITED STATES

## Abstract

Overweight and obesity have become global concerns in developed and developing countries due to their rise in recent years and their association with the prevalence of non-communicable diseases including diabetes, hypertension and cardiovascular diseases. In fact, it is estimated that roughly 39% of adults worldwide are overweight and 13% are obese. Ecuador is an example of a developing country concerned with the overweight and obesity problem, where it is estimated that 30% of children, 26% of teenagers and 63% of adults are either overweight or obese and where 1 in 4 deaths are attributed to chronic diseases. To address the overweight and obesity problem via the promotion of healthy eating habits, in 2013 the country approved technical regulation for the labelling of packed processed food products. The regulation included a mandatory traffic-light (TL) supplemental nutritional information labelling system to be displayed on the package of all processed foods for sale in the country. This new labelling system displays a traffic light panel for the product content of sugar, fat and salt in addition to the traditional nutrient declaration label. The objective of this paper was to evaluate the effect of the TL supplemental nutritional information on consumers’ buying behavior in Ecuador. More specifically, we concentrated on the purchasing behavior of carbonated soft drinks. For our analysis, we used monthly aggregated purchase data (total expenditures, quantities and average prices) of carbonated soft drinks from January 2013 to December 2015 obtained from Kantar World Panel—Ecuador. We estimated a non-linear Almost Ideal Demand System where we model the demand for high sugar and low sugar carbonated soft drinks. We found that the introduction of the traffic light supplemental nutrition labelling did not have the expected effect of reducing purchases of carbonated soft drinks during its first year of implementation, especially those high in sugar. Additionally, we found that lower income-status households tend to spend more on and consume more calories from CSD than households with higher socio-economic status. Finally, we identified that over time purchases of high sugar soft drinks decreased while purchases of low and no sugar soft drinks increased. Beyond our contribution of evaluating the effect of the traffic light on the purchases of carbonated soft drinks, we also estimated price and income elasticities of carbonated soft drinks which can be useful in the evaluation of fiscal policies.

## Introduction

According to the World Health Organization, in 2014 roughly 39% of adults worldwide were overweight and 13% were obese [1]. These problems are not only prevalent in high-income countries; many low- and middle-income countries are now also experiencing problems with obesity. For example, according to the 2012 Health and Nutrition National Survey (HNNS) conducted in Ecuador, considered by the World Bank a middle-income country, 30% of school-aged children, 26% of teenagers and 63% of adults are either overweight or obese as result of a diet high in calories and low physical activity [2]. In addition, the prevalence of chronic diseases associated with overweight and obesity such as diabetes, hypertension, and cardiovascular disease are considered high in the country and estimated to be related to about 1 in 4 deaths [2].

The 2012 HNNS found that the Ecuadorean diet includes excessive amounts of rice, palm oil and dairy, and low amounts of fruits, vegetables and legumes, which results in an intake of refined carbohydrates and saturated fats above international recommendations. Additionally, the HNNS identified high intake of sugary beverages by the population. For example, 82% of teenagers reported consuming carbonated soft drinks (CSD) regularly. Because of these findings, the report recommended a comprehensive front-of-package labelling system to help consumers better interpret the content of fat, sugar and salt in processed foods. Shortly after, in November of 2013, the Ecuadorean Ministry of Public Health issued the technical regulation for the labelling of packed processed food products, which aims to address the prevalence of chronic diseases associated with overweight and obesity via the promotion of healthy eating [3].

The Ecuadorean labelling of packed food products regulation established the inclusion of a traffic-light (TL) like graphical system in the package of processed foods for sale in the country for both domestic and imported food products. The system is intended to provide consumers with easy to interpret nutritional information related to a food product’s contents of sugar, fat and salt beyond the information already included in the nutrition facts labels regulated by the *Codex Alimentarius*. Medium and large food companies were required to comply with the regulation before August 29^th^, 2014, and small companies before November 29^th^, 2014 [3].

Because the policy is relatively new, the literature evaluating its impact on the purchasing habits of Ecuadorean consumers is very limited. No previous study has evaluated the impact of the policy using actual households’ food purchases data. Therefore, the objective of this paper is to evaluate the impact of the TL nutritional information system in the buying habits of Ecuadorean consumers. More specifically, we focus on the impact of TL on the buying habits of CSD given their high level of consumption in the country and the Latin American region [2, 4]. Moreover, the main health concern with the consumption of CSD is their sugar content. The focus on only one nutrient, as we will discuss later, simplifies the analyses and interpretation of results given data limitations.

This paper also contributes to the international nutritional policy literature by expanding the limited body of studies that empirically evaluates the effectiveness of nutritional labelling aimed at changing the buying and consumption habits of the population toward *healthier* food products [5, 6]. To the best of our knowledge, this is the first study to evaluate a supplemental nutritional labelling policy implemented at the national level. This is important since other countries, such as Chile, have adopted similar supplemental nutritional labelling policies [7]. Another important contribution of this study is the estimation of CSD products’ price and expenditure elasticities which can be useful for the evaluation of fiscal policies.

### Traffic-light nutritional labelling in Ecuador

**Fig 1:**
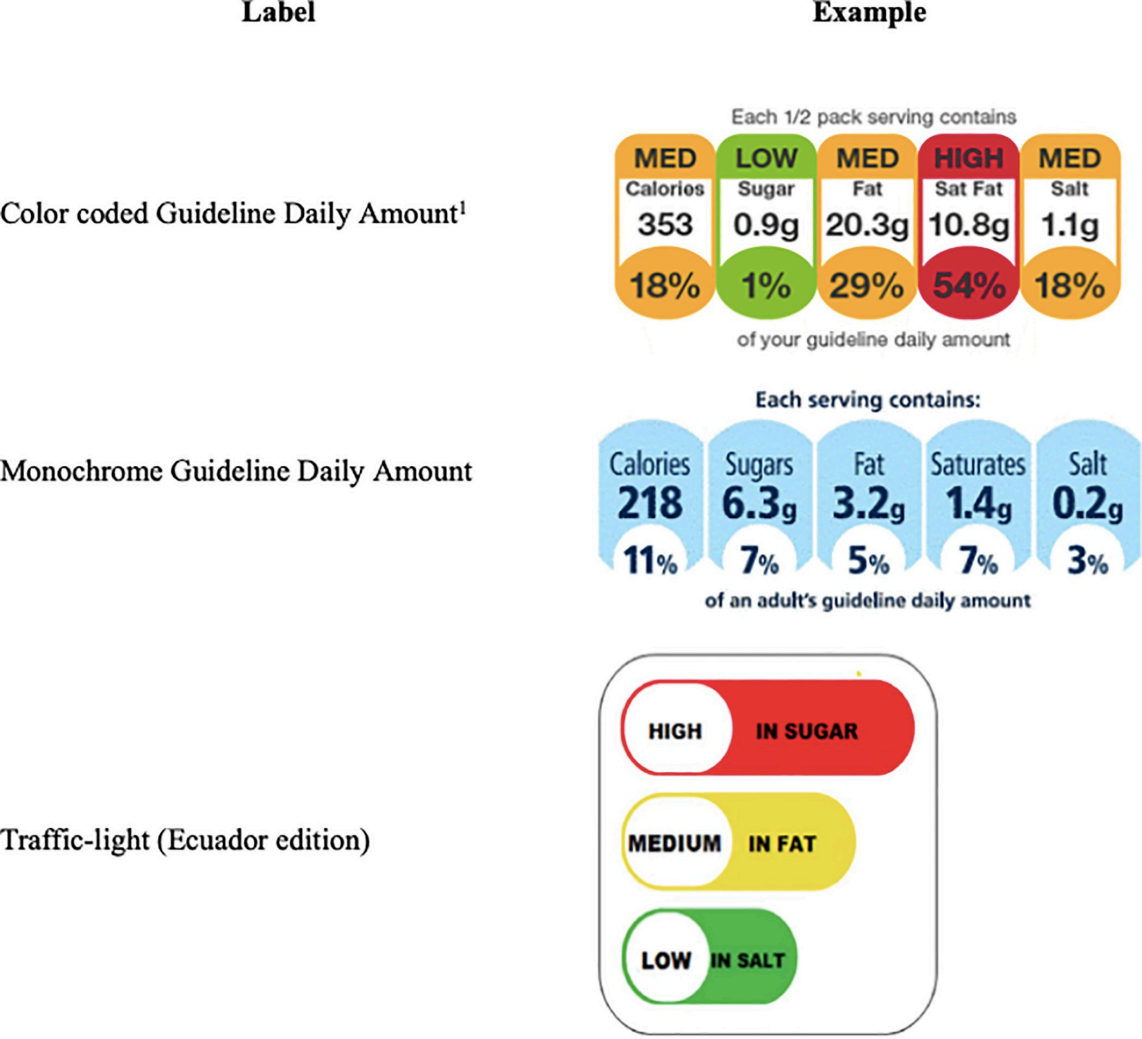
Supplemental nutritional information labels.

Source: Own with images from www.pacakingnews.co.uk and Freire *et al*. [18].

There are two main types of nutritional labels for package products: 1) nutrient declaration/facts labels, and 2) supplementary nutrition information labels. The nutrient declaration label is the standard label that can be found in any processed food product that shows the serving per container and the nutritional content per-serving and the percentage daily value based on a 2,000 calorie diet. It is intended to provide consumers with a profile of the nutrient composition of the food product and its inclusion is mandatory in many countries including Ecuador [3, 8]. Supplementary nutrition information labels, as their name suggests, are intended to help consumers better interpret the nutrient declaration label to improve their understanding of the nutritional content of food products. There are two types of supplementary nutrition labels: nutrient specific and summary systems [9]. Nutrient specific supplementary nutrition labels indicate information on a few key ingredients whereas summary systems provide an overall nutrient score (e.g., a number or stars)[9]. The TL label is a nutrient specific supplementary nutrition label. In contrast to the TL label adopted in other regions which contains information on 5 nutrients [9], the TL label in Ecuador only denotes the content of 3 ingredients: sugar, fat and salt [3] (Fig 1).

The Ecuadorian labelling regulation considers four levels of concentration for each of the three nutrients: *low*, *medium*, *high* and *it does not contain* (Table 1). For each nutrient, a green light and the word *Low* are used if the concentration is considered low. Similarly, a yellow light and the word *Medium* and a red light and the word *High* are used for medium and high concentrations of the nutrients, respectively. If the food product does not contain a nutrient, no traffic light is used but a “*it does not contain*” message is added before the name of the nutrient that is not present.

**Table 1:** Nutrient content and traffic light color.

Component	Level		
	‘Low’ concentration (Green)	‘Medium’ concentration (Yellow)	‘High’ concentration (Red)
Total fat	≤ 3gr/100gr or ≤ 1.5gr/100ml	between 3 and 20gr/100gr or between 1.5 and 10 gr/100ml	≥ 20gr/100gr or ≥ 10gr/100ml
Sugars	≤ 5gr/100gr or ≤ 2.5gr/100ml	between 5 and 15gr/100gr or between 2.5 and 7.5 gr/100ml	≥ 15gr/100gr or ≥ 7.5gr/100ml
Salt (Sodium)	≤ 120 mg/100gr or ≤ 120 mg/100ml	between 120 and 600mg/100gr or between 120 and 600 mg/100ml	≥ 600mg/100gr or ≥ 600 mg/100ml

Source: Ecuadorian technical regulation RTE INEN 022 (2R) [3]

According to the technical regulation [3], the presence of the TL label is in addition to the nutrient declaration label, it can be placed in the front or back of the product’s package and its size must be commensurate to the size of the chosen panel (between 15 and 32%). Given the rounded shape of CSD containers, the TL label is placed on the “side” of the bottle whereas the “front” contains the product’s name.

### Literature review

There is an abundant body of literature evaluating the acceptability by consumers and efficacy of supplementary nutrition labels at helping consumers identify healthier food alternatives in controlled and experimental environments, but very limited literature empirically evaluating its effect on actual consumer behavior.

Overall, the literature suggests that TL labelling is more effective than other types of supplementary nutrition information labels in helping consumers identify healthier products. It has been found that consumers are more likely to identify healthier products when the TL labelling is used compared to GDA labelling [10, 11]. Additionally, consumers are able to better interpret the nutritional information when TL is used relative to GDA labelling and also when no supplementary nutrition information is provided [12]. Some authors also argue that TL labelling not only helps consumers better identify the healthiness of the product but also reduces the complexity of the decision making because of its simplicity [13]. While consumers may understand the TL, evidence of its effectiveness on intended or hypothetical purchasing decision is mixed [14, 15].

With respect to the literature evaluating the effect of the TL labelling on actual consumer buying behaviour, Sacks et al. evaluated retailer sales of ready meals and sandwiches in the United Kigdom after the introduction of a voluntary TL labelling on the package of the retailer’s own brands [6]. While supplementary nutrition information labelling is not required in the United Kingdom, the Public Ministry of Health recommends its use. During the period of the study, products with and without TL labels were available to consumers. To evaluate the effectivenes of TL labelling at promoting the purchases of healthier alternatives, the study assessed the association between the change in sales after the introduction of the label and the healthiness of the products according to the color of the TL labels. The results showed no significant association between these two variables. Another study conducted in Australia evaluated online sales of 53 food products with and without the TL label displayed on the product website. The study results also suggested no association between the change in sales of the products after the introduction of the labels and their healthiness [16]. A limitation of both studies is that they were conducted over very short periods of time[6, 16]. Another retail study conducted in the United States, found that in-store TL labelling when combined with financial incentives modestly reduced the consumption of sugar sweetened beverages after 5 months of the introduction of the TL [17].

Finally, we only identified three studies related to the use of the TL labelling in Ecuador [18, 19, 20]. These studies found that whereas consumers indicate they know about and understand the TL label, they also acknowledge its presence does not influence their purchasing decisions [19, 20]. None of these studies evaluated the effect of the TL labelling on actual purchasing behavior.

### Conceptual framework

According to neoclassical consumer theory, consumers maximize utility from the consumption of goods and services subject to a budget constraint. The utility function represents consumer preferences which are based on knowledge and information they have available (i.e., consumer´ information set). The introduction of a policy such as the TL labelling makes available new information that consumers can use in their decision-making process as part of their information set. As a result, the demand curve of soft drinks after the introduction of the TL may not be same as the one before the introduction of the TL labelling [21]. Therefore, information in general, and TL labels particularly, can both shift and rotate the demand curve (see also Teisl, Bockastael, and Levy’s 2001 for an alternative theoretical formulation [22]), as they change consumer´s willingness to pay (WTP) for a product. A shift in the demand curve correspond to the case when the effect on WTP for a product due to information is the same for all consumers. On the other hand, rotation in the demand curve corresponds to the case where the effect in WTP values differs across consumers [23, 24]

**Fig 2:**
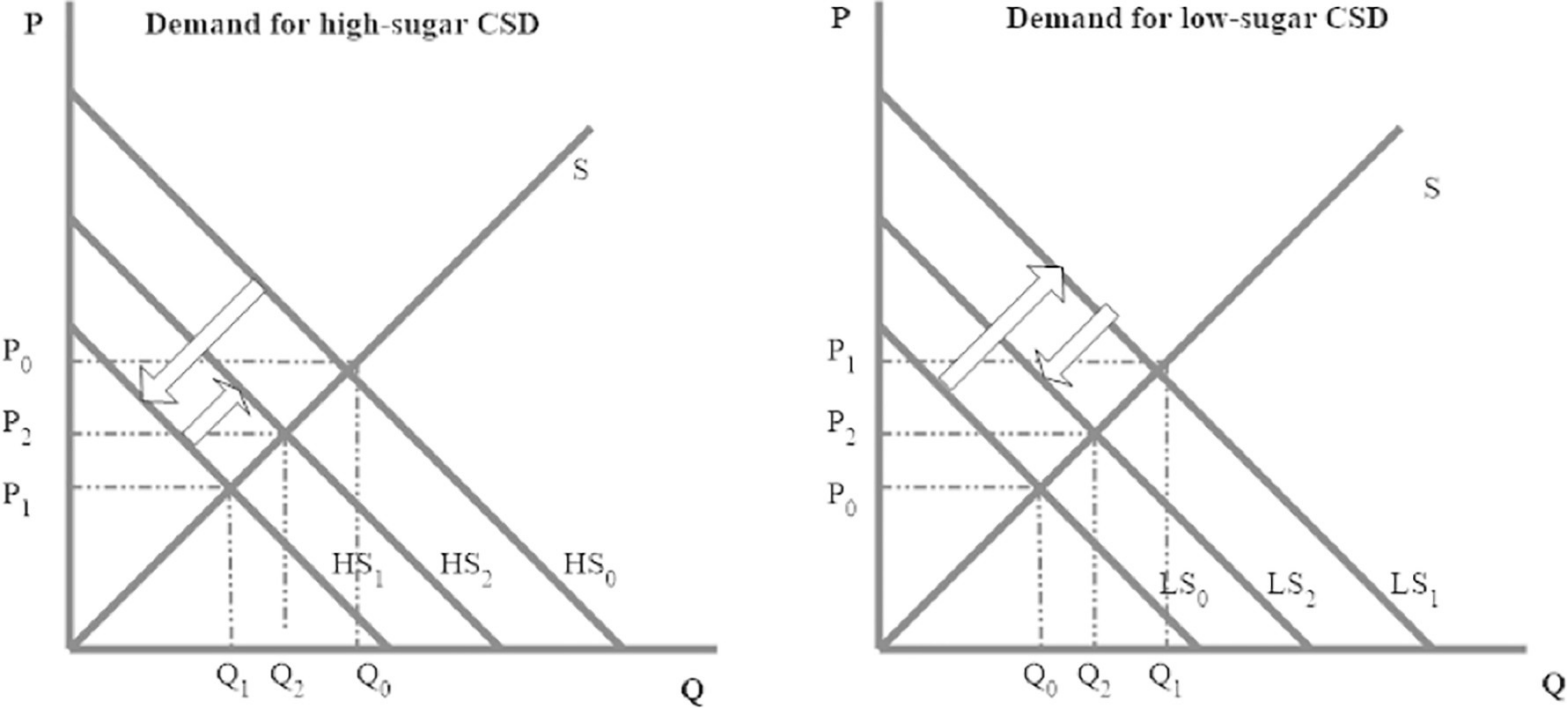
Shifts in the demand for carbonated soft drinks.

For illustration purposes, consider the market demands for high-sugar CSD, for low-sugar CSD (Fig 2) and shift effects only. TL labelling aims to reduce the consumption of sugar from soft drinks by inducing a downward shift in the demand for high sugar CSD (from HS_0_ to HS_1_) and an upward shift in the demand for low sugar CSD (from LS_0_ to LS_1_).

The shifts cause changes in the equilibrium quantities and prices in both markets. The downward shift from HS_0_ to HS_1_ in the high-sugar soft drinks market causes a reduction in the equilibrium quantity demanded from Q_0_ to Q_1_ and a decrease in the equilibrium price from P_0_ to P_1_. Similarly, the upward shift from LS_0_ to LS_1_ causes an increase in the equilibrium quantities and prices for low sugar beverages. If, as it should be expected, high- and low-sugar CSD are substitutes, the increase in the price of low-sugar CSD shifts upward the demand curve for high-sugar CSD and the decrease in the price of high-sugar CSD shifts downward the demand curve for low-sugar CSD; thus, both curves would tend to move the curves towards their original positions. The final effect of the policy is thus dependent upon the magnitude of the demand shifts as well as supply and demand relations.

For simplicity, the aforementioned theoretical model only considers the markets for two aggregate CSD products (high sugar and low sugar) but it highlights the expected market effects of TL labelling as well as the importance of considering market interdependencies into the analyses. The empirical model used in this study takes into account several CSD products as well as other food products and uses micro-level data to evaluate the final impact of the TL policy on the demand for CSD products. Our empirical approach also allows us to evaluate the effect of the TL on the slope of the demand curves. The information in the label would be expected to make high sugar CSD more sensitive to changes in the own price (i.e., steeper) and low sugar CSD less sensitive to changes in the own price (i.e., flatter).

## Materials and methods

### Data

This study uses monthly CSD purchase data (volume in litters (L) and monetary value in US$) from a panel of Ecuadorean households from January 2013 to December 2015. This time frame allows us to observe consumer purchases for 20 months before and 16 months after the final deadline for compliance with TL labels.

The data was obtained from Kantar World Panel Company, which collects weekly food purchase data from a panel of 1,646 households. Households in the panel are visited by an interviewer once a week to record purchases using the bar code scan method. This method allows the collection of both product brand information and purchased volumes. Prices are obtained from the corresponding receipts or from diaries kept by the person in charge of household purchases. Twenty-two percent of consumers in the panel are from the capital Quito, located in the Mountain region, and 27% from Guayaquil, the second largest city located in the Coastal region. Seventeen percent of the sample comes from participants located in other cities in the Mountain region and 34% from participants located in other Coastal region cities. Ninety-one percent of the urban population and 55% of the total population live in the regions represented in the panel. According to Kantar, their data collection design ensures that the data is representative of the population of shoppers in these regions. The Panel also provides information representative of three groups of households aimed to represent different socio-economic status groups: *high* and *middle-high* socio-economic status group (7% of the population), *middle clas*s (27% of the population), and *low* and *very-low* socio-economic status group (66% of the population). Kantar World Panel classifies households based on their ability to satisfy their basic needs. Households are considered as *high* socio-economic status if they satisfy all their basic needs and can afford some luxuries. Households that can completely satisfy all their basic needs are classified as *middle-class*. If households can barely satisfy or are not able to satisfy their basic needs, they are classified as *low* and *very-low* socio-economic status households, respectively [25].

The data set contains purchase information of 13 food groups and 17 drink groups including the CSD group. The CSD group contains purchase information on 23 brands of CSD from 9 different companies and an ‘all- other-CSD’ category. Although the data was collected at the household level, only aggregate monthly level data for each socio-demographic group was made available to us and used in the analyses. Finally, to transform monthly aggregated data of the panel to per-capita values for all CSDs groups, we used population estimates from the World Bank [26]. Using Coca Cola as an example, Kantar provided us with and estimated total monthly purchases of this CSD among all high, middle and low-income status households in Ecuador (separate data for each household group). World Bank population estimates of households on each socio-economic status group were then used to calculate monthly per-capita consumption of regular Coca Cola in each household group. Thus, final monthly data for the analyses included estimated per-capita purchases of regular Coca Cola for high, middle and low-income status households for a total of 108 observations (36 months by 3 socio-economic levels) Since we have 20 months before the implementation and 16 after, we have a total of 60 pre-policy observations and 48 post-policy observation.

### A model for the demand for carbonated soft drinks

To evaluate the effect that the TL has on the demand for CSD we used the non-linear Almost Ideal Demand System (AIDS) [27]. The use of a demand system approach allows us to explore the differential impact of the policy across CSD groups (high versus low sugar) of different brands. The demand system approach allows for the use of economic theory in the simultaneous estimation of the equations, which results in gains in efficiency of the estimated parameters [28, 29].

The AIDS demand system specification is

$$w_{iht}=\alpha_{ih}+\sum_{j=1}^{5}\gamma_{ij}\;\ln\;p_{jht}+\beta_{i}\;\ln\;\left(\frac{E_{ht}}{P_{ht}}\right)+\delta_{ih}\prime z_{iht}+\varepsilon_{ikt},$$

where *i* the index for soft drink groups, *h* is the index for household socio-economic status group, and *t* is the time period. *w_iht_* are the budget shares, *p_jht_* are soft drink prices where *j* is the prince index corresponding to each soft drink group, *E_ht_* is total per-capita monthly expenditures in food and drinks, *P_ht_* is a price index, **z_iht_** is a vector of other factors affecting demand including a time trend, socio-economic status of the household, quarterly dummy variables, and a dummy variable for the introduction of the TL labelling policy. The *α_i_′s,γ_ij_′s, β_i_′s*, and **δ_i_′*s*** are model parameters. Details about the construction of prices are included in S1 Appendix.

Our demand system consists of 5 linear equations (i = 1,2,…,5). The first equation of the demand system corresponds to the CSD with the highest market participation, which is Coca-Cola with 57.9% average market share during the period of observation. The second equation corresponds to its direct dark colored and high sugar competitors, which are Pepsi and Big-Cola with a combined 9.58% average market share. The third equation corresponds to the low- and no-sugar CSD with 3.2% of combined average market share. The fourth one is composed of all the other high sugar CSD. The National Agency for Regulation, Control and Sanitary Surveillance of Ecuador (ARCSA) provided us with a data set containing the formulation of most carbonated soft drinks before and after August of 2014. We used this data to categorize the CSD into high- and low-sugar. Finally, we include an equation for a numeraire good that includes all other foods and beverages consumed by the households in the panel.

Although a larger demand system could be considered, the lack of degrees of freedom precludes the estimation of such a system. Coca-Cola was included by itself due to its high market share. The other CSD groups were chosen based on both market share importance as well as classification as high or low sugar products (see Table 2).

**Table 2:** Brands per category and average expenditures and quantities purchased before and after introduction of the policy.

	Mean per-capita monthly expenditures (U.S.$)*		Mean per-capita monthly quantity purchased (L)		
Category	Before	After	Before	After	Brands/types of product
Coca-Cola	0.67	0.58	0.99	0.88	Coca-Cola
Dark colored high sugar	0.07	0.08	0.19	0.18	Pepsi and Big-Cola
Low- and non-sugar	0.04	0.04	0.01	0.03	Coca-Cola light, Coca-Cola zero, Sprite zero, Inca-Kola and Barrilitos-O-Key.
All other high sugar sodas	0.31	0.28	0.53	0.54	Coca-Cola life, Fanta, Frioravanti, 7up, Mas, Kola gallito, Oro, Tropical, Quintuples, Orangine, Fox Cola, Fruit and all other.
Total	1.09	0.98	1.72	1.63	All

*The period before corresponds from January 2013 to August 2014 (on August 29th all medium and large companies were required to comply with the TL labelling) and the period after corresponds from September 2014 to December 2015.

Since the estimated model uses as dependent variable expenditure shares, model parameters were subsequently used to calculate: 1) the impact of the explanatory variables on CSD expenditures and quantities demanded (i.e., marginal effects), with special emphasis on the effect of the TL light label, 2) CSD price and expenditure elasticities, and 3) the effect of TL light label on the consumer responses to price changes. All the formulas used for the calculations are included in S1 appendix.

Estimation of the demand system parameters was carried out using Seemingly Unrelated Regression procedures with the proc model procedure in SAS®. The last equation corresponding to the numeraire good was dropped from the demand system, and its parameters recovered using the adding up constraint. Homogeneity and symmetry demand restrictions were also imposed in the demand system [30]. To account for potential heteroscedasticity, autocorrelation of the errors, and the clustering nature of the data (we have three observations per period corresponding to the three socioeconomic status groups) standard errors for parameters, marginal effects and elasticities were estimated using a moving block bootstrapping procedures with 500 repetitions [31, 32].

## Results and discussion

This section is organized as follows. To gain a better understanding of the carbonated soft drinks market in Ecuador, we first present and discuss descriptive statistics. Demand estimation results are discussed subsequently and finally we discuss the estimated effects of the traffic light on the demand for CSD as well as other demand shifters.

### Descriptive statistics

The CSD market is dominated by one brand, Coca-Cola, which accounts for approximately 57% of CSD purchases. Low- and non-sugar CSD alternatives represent only a small fraction of the CSD market, about 3.22%. Prices remained relatively constant during the observed period with mean price of $0.60/L. and an average coefficient of variation of 14%.

On average, Ecuadoreans purchased 1.67 L per-capita per month (LPCM) of CSD during the observed period ranging from 1.45 to 2.07 LPCM. Before the introduction of the TL, CSD purchases averaged 1.71 LPCM, while after the introduction of the TL it averaged 1.62 LPCM. However, these levels of quantities purchased are not statistically different at *α* = 0.05 (*t* = 2.03;*p* = 0.08) It is also important to emphasize that these data only represents purchases for consumption at home, and that actual per-capita purchases is higher when purchases for consumption outside the home is taken into consideration (Table 3).

**Table 3:** Descriptive statistics of carbonated soft drinks monthly quantity purchased and prices (January 2013- December 2015).

Brand		Volume—Liters per-capita				Price—US$ per-liter			
	N	Mean	Std. Dev.	Min.	Max.	Mean	Std. Dev.	Min.	Max.
Coca-Cola	108	0.958	0.150	0.639	1.344	0.651	0.033	0.496	0.769
Coca-Cola Life	108	0.001	0.003	0.000	0.015	0.868	0.108	0.696	1.110
Coca-Cola Zero	108	0.008	0.008	0.000	0.028	0.695	0.099	0.134	0.925
Coca-Cola Light	108	0.017	0.012	0.000	0.053	0.759	0.090	0.576	1.400
Fanta	108	0.055	0.013	0.029	0.095	0.663	0.036	0.591	0.841
Fioravanti	108	0.106	0.025	0.054	0.155	0.635	0.029	0.572	0.707
Sprite	108	0.136	0.031	0.077	0.217	0.660	0.026	0.609	0.834
Sprite Zero	108	0.006	0.06	0.000	0.024	0.689	0.126	0.097	1.235
Inca Kola Regular	108	0.026	0.018	0.003	0.102	0.623	0.055	0.427	0.757
Pepsi	108	0.065	0.023	0.023	0.112	0.562	0.035	0.515	0.732
7Up	108	0.022	0.008	0.006	0.047	0.577	0.048	0.487	0.771
Mas	108	0.018	0.007	0.006	0.035	0.543	0.045	0.391	0.764
Kola Gallito	108	0.016	0.009	0.003	0.046	0.532	0.060	0.391	1.005
Big Cola	108	0.071	0.061	0.002	0.202	0.531	0.054	0.443	0.833
Oro	108	0.006	0.010	0.000	0.037	0.525	0.065	0.432	0.833
Tropical	108	0.059	0.015	0.032	0.099	0.572	0.029	0.500	0.650
Manzana	108	0.042	0.012	0.017	0.076	0.562	0.034	0.437	0.642
Quintuples	108	0.012	0.005	0.002	0.035	0.599	0.065	0.453	0.853
Oranguine	108	0.005	0.003	0.000	0.024	0.499	0.069	0.333	0.756
Fox Cola	108	0.002	0.002	0.000	0.008	0.504	0.150	0.188	1.369
Barrilitos-O-Key	108	0.002	0.003	0.000	0.012	0.484	0.175	0.267	1.126
Fruit	108	0.001	0.001	0.000	0.005	0.529	0.156	0.272	1.111
Others	108	0.002	0.002	0.000	0.010	0.628	0.250	0.075	1.747

**Fig 3:**
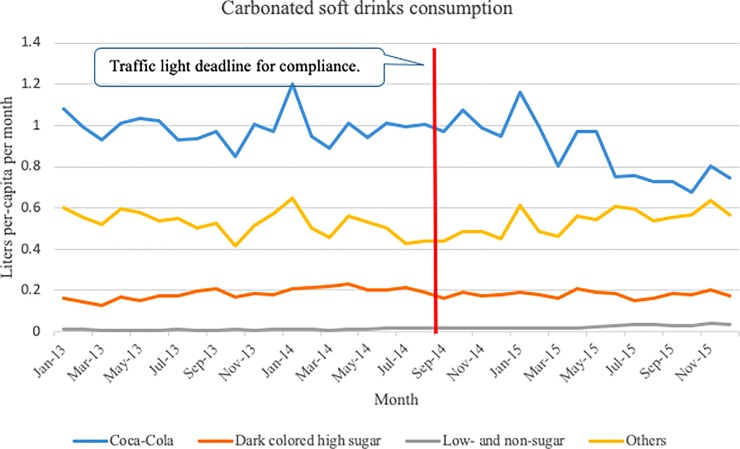
Carbonated soft drinks purchases.

Overall, purchases of Coca-Cola exhibits a downward trend, which is evidenced by average purchases of 0.98 LPCM before the introduction of the TL and average purchases of 0.87 LPCM after the introduction of the TL, while at the same time purchases for all other high sugar CSD and low- and non-sugar CSD exhibit a moderate increase. Particularly, purchases of low- and non-sugar CSD averaged 0.01 and 0.03 LPCM, before and after the introduction of the TL, respectively. Purchases of dark colored high sugar sodas remained constant over time (Fig 3).

The use of a demand system approach allowed us to not only evaluate the effect of the introduction of the TL label on households’ CDS purchases; but also households’ CSD purchase responses to changes in prices and total expenditures (i.e., price and expenditures elasticities of demand), differences in consumption by socio-economic status, and changes in consumption due to seasonal patterns or time trends. The rest of our results are presented as follows: 1) price and expenditure elasticities, 2) the effect of socio-economic status and trends on purchases, 3) the effect of the TL on quantity demanded and consumer and price elasticities (which is the main objective of our research), and 4) robustness analyses.

### Demand system estimation results

#### Price elasticities of demand and expenditure elasticities

Consistent with demand theory all own-prices elasticities were negative. The magnitude of the own-price elasticities indicates that all soda drinks but Coca-Cola are price elastic. There is only one previous published study that estimated elasticities for sugar sweetened beverages (SSB) in Ecuador [33]. This study found the own-price elasticity of SSB to be elastic, ranging from -1.328 for the lowest quintile of the population to -1.201 for the highest quantile. However, it is important to mention that the SSB group consider included several types of sugary beverages including CSD [33]. In Mexico, estimates of the price elasticity of soft drinks as an aggregate product range from -0.6 to -1.6 [34]. It is expected to find higher own-price elasticities at a lower level of aggregation.

Regarding cross price elasticities between high and low sugar CSD products we find that in some cases they are substitutes and in other cases they can be complements. For example, low- and non-sugar CSD are substitutes for Coca-Cola and dark-colored high-sugar CSD and vice versa. On the other hand, low-and non-sugar CSD are complements with all other high- sugar sodas. Coca-Cola and dark colored high sugar sodas are found to be complements. The *numeraire* good (all other foods) is a complement of all the high sugar CSD categories as all the cross-price elasticities had a negative sign (see Table 4). Similarly, all the high sugar CSD are complements to the *numeraire* good. The complementarity between Coca Cola and Dark colored high-sugar drinks was an unexpected result, but it is not uncommon to find counterintuitive results with cross price elasticities. It is also well documented that cross-price elasticities are difficult to identify when using flexible demand systems [35, 36]. This means that they are difficult to estimate with precision (i.e., they are highly variable).

**Table 4:** Marshallian price and expenditure elasticities.

	Coca-Cola	Dark colored high-sugar	Low- and non-sugar	All other high sugar sodas	All other foods	Expenditure elasticities
Coca-Cola	-0.597	-0.315	0.258	0.447	-0.435	0.641
	(0.487)	(0.081)	(0.090)	(0.256)	(0.239)	(0.115)
Dark colored high-sugar	-2.464	-1.955	0.417	3.463	-0.252	0.791
	(0.627)	(0.449)	(0.292)	(0.797)	(0.570)	(0.210)
Low- and non-sugar	4.369	0.905	-2.806	-3.211	0.755	-0.012
	(1.569)	(0.651)	(0.643)	(1.372)	(1.213)	(0.420)
All other high sugar sodas	0.940	0.933	-0.402	-1.244	-0.767	0.540
	(0.542)	(0.214)	(0.167)	(0.627)	(0.365)	(0.148)
All other foods	-0.021	-0.002	0.000	-0.015	-0.979	1.018
	(0.007)	(0.002)	(0.002)	(0.005)	(0.009)	(0.004)

Standard errors in parenthesis.

Also, consistent with demand theory, all expenditure elasticities for high sugar CSD were positive. The expenditure elasticities suggest that all high sugar CSD are necessary goods (see Table 4). A negative sign was found for the expenditure elasticity for low- and non-sugar CSD but this elasticity was not estimated very precisely. Paraje [33] also found that SBB are necessary goods for the average Ecuadorian consumer although they also found that they are luxury goods for low-income households. The parameter estimates of the demand system are available in S2 Appendix.

#### Effects of socio-economic status and time series components

The differences on mean expenditures, expressed in US$/month per-capita, and mean quantities, expressed in LPCM (Tables 5 and 6, respectively) estimated by the socio-economic status dummy variables suggest that households with high and medium socio-economic status purchase less Coca-Cola, dark colored high- sugar all other high-sugar CSD and more low- and non-sugar CSD than households with low socio-economic status (the baseline category). In Tables 5 and 6, each of the rows represents an equation estimated in our demand system. One for Coca-Cola, one for dark-colored high-sugar substitutes of Coca-Cola, low- and non-sugar CSD all other high-sugar and one for a numeraire good which includes all other foods and drinks. The magnitude of this difference in purchases is quite important. The quantity purchased of high sugar CSD by households in the high socio-economic status is 0.476 LPCM lower than the quantity purchased of CSD by households in the low and very low socio-economic status (which is equivalent to about 30% of average per capita quantity purchased of high-sugar CSD in the country during the study period). Similarly, the estimated difference in the quantity demanded of low- and non-sugar CSD by these income groups (0.023 LPCM) represents about 95% of average quantity purchased of low- and non-sugar CSD during the period of observation. Meanwhile, the medium socio-economic status group also purchases less high sugar CSD and more low and non-sugar CSD than the low socio-economic status group, but the magnitude of this difference is much smaller than the one observed in the high socio-economic status group. Overall, the socio-economic status coefficient shows that lower income-status households tend to spend more on and consume more calories from CSD than households with higher socio-economic status.

**Table 5:** Effects of the demand shifters on mean expenditures ($ per-capita per month).

	High socio-economic status	Medium socio-economic status	Time trend	Traffic light labelling	1^st^ quarter	2^nd^ quarter	3^rd^ quarter
Coca-Cola	-0.088	0.015	-0.007	0.090	0.072	0.035	-0.019
	(0.032)	(0.025)	(0.001)	(0.019)	(0.016)	(0.014)	(0.014)
Dark colored high-sugar	-0.117	-0.082	0.001	-0.015	0.004	0.003	-0.003
	(0.008)	(0.006)	(0.000)	(0.006)	(0.004)	(0.004)	(0.003)
Low- and non-sugar	0.023	0.009	0.002	-0.005	-0.004	-0.001	-0.002
	(0.007)	(0.006)	(0.000)	(0.005)	(0.003)	(0.003)	(0.004)
All other high sugar sodas	-0.097	-0.013	0.000	0.010	0.031	0.022	-0.017
	(0.020)	(0.016)	(0.001)	(0.014)	(0.012)	(0.009)	(0.009)

Standard errors in parenthesis. Note: Rows in the table include marginal effects corresponding to each of the four demand equations estimated in the system. The baseline category for socio-economic status effects is the low socio-economic status. Baseline category for the effects of quarters is the 4^th^ quarter.

**Table 6:** Effects of demand shifters on mean quantities (L per-capita per month).

	High socio-economic status	Medium socio-economic status	Time trend	Traffic light labelling	1^st^ quarter	2^nd^ quarter	3^rd^ quarter
Coca-Cola	-0.132	0.022	-0.011	0.135	0.109	0.053	-0.029
	(0.048)	(0.038)	(0.002)	(0.028)	(0.025)	(0.021)	(0.021)
Dark colored high-sugar	-0.193	-0.135	0.001	-0.025	0.006	0.004	-0.004
	(0.013)	(0.010)	(0.000)	(0.009)	(0.006)	(0.006)	(0.005)
Low- and non-sugar	0.035	0.013	0.002	-0.008	-0.006	-0.001	-0.004
	(0.011)	(0.009)	(0.000)	(0.007)	(0.005)	(0.005)	(0.005)
All other high sugar sodas	-0.151	-0.020	0.000	0.016	0.048	0.035	-0.026
	(0.031)	(0.025)	(0.001)	(0.022)	(0.018)	(0.014)	(0.014)

Standard errors in parenthesis. Note: Rows in the table include marginal effects corresponding to each of the four demand equations estimated in the system. The baseline category for socio-economic status effects is the low socio-economic status. Baseline category for the effects of quarters is the 4^th^ quarter.

During the period of observation, we observed an overall downward trend in the quantity purchased of (-0.010 LPCM) and expenditures (-$0.008/month) on high-sugar CSD (due mainly to the decrease in purchases of Coca Cola) and an upward trend in the quantity purchased of (0.002 LPCM) and expenditures ($0.002/month) on low- and non-sugar CSD. These trends might reflect overall long-term trends in the demand of high and low sugar CSD. We also identified some seasonality patterns in the purchases of Coca-Cola and all other high-sugar CSD: higher purchases of these products in the first and second quarter relative to the fourth quarter. These seasonality patterns may reflect differences in demand across seasons related to the Carnival and Holy Week festivities that take place in the country during the first and second quarters.

#### The effect of the ‘Traffic-Light’ label in the quantity demanded and own price elasticities of CSD

The effect of the TL label on CSD purchases can be evaluated using the estimated effects of the TL label on the individual CSD categories used in the demand model as well as more aggregate CSD categories (all CSD and high sugar CSD). When analyzing the individual estimated effects of the TL policy for the quantities demanded for the individual CSD categories, all the estimated effects, but the effect on dark colored high sugar CSD, are not statistically different than zero at the 5% level when using one tail tests. Purchases of dark-color high sugar CSD was estimated to have decreased by 0.025 LPCM. One tailed tests are used as it was expected for the policy to decrease the overall purchases of CSD and high sugar CSD, and increase purchases of low o non-sugar CSD [37]. One tail tests provide gains in power which is especially important given our small sample size [38].

The aggregated estimated effect on quantity demanded of all high sugar CSD (0.127 LPCM) as result of the presence of the TL labelling is, contrary to expectations, positive although it is also not statistically different from zero using a one tail test. Finally, the total estimated across all CSD is also positive (0.119 LPMC capita) and also not statistically different from zero using a one tail test.

With regard to the effect of the TL label on the own price elasticities, all the estimated effects are small in relative terms (less than 10% of the own price elasticities), and non-significant (p values > 0.14). For example, the estimated effect of the TL label on the own price elasticity of Coca Cola is -0.0395 which would suggest that the demand for Coca Cola has become slightly more own price elastic; however, the estimated effect is also non-significant (p = 0.567). Thus, we did not find evidence that the introduction of the TL label had changed CSD demand responses to changes in their own prices.

Freire et al. [20] argue that the potential impact of the TL in Ecuador has been limited by several factors including effective promotional efforts. However, it is also possible that the TL system does not influence consumer behavior as has been found recently in some experimental studies [13, 16, 17, 39].

### Robustness analysis

Several robustness checks were carried out to evaluate the sensitivity of the results to several assumptions underlying the analyses. First, in addition to the non-linear AIDS model, we estimated Rotterdam and Exact Affine Stone Index (EASI) models [30, 40, 41]. Second, we used the Zivot-Andres Unit Root Test to identify potential break points in the demand for different CSD categories that could be attributed to the TL labelling. The results suggested two potential break points, at point 18 (June 2014) and at point 24 (December 2014). Therefore, we tested three starting points for the TL dummy variable, point 18 (a potential breakpoint if all companies complied before the deadline), 20 (deadline for compliance for medium and large companies) and 24 (which coincides with the deadline for compliance for small companies). Since the effect of the TL labelling was very similar, at all starting points we decided to keep it at December 2014 in our final analysis, thus allowing us to ensure all CSD available to consumers displayed the TL nutritional labelling. Table 7 summarizes the overall effect of the TL labelling on the demand for all high-sugar CSD in different model specification and potential break points of the TL dummy. In all cases, we found an overall increase in the demand for high sugar CSD after the introduction of the TL label. All the other results related to the effect of TL on total CSD quantity demanded and own price elasticities were also similar (to those found in the AIDS model) across model specifications and break points.

**Table 7:** Estimated effect of the introduction of the TL label on the demand for high sugar CSD (L/month per-capita).

	Potential break points in the demand for CSD		
Model	18	20	24
AIDS	0.084 (0.040)	0.127 (0.037)	0.058 (0.036)
Rotterdam	0.006 (0.052)	0.009 (0.054)	0.003 (0.105)
EASI	0.096 (0.043)	0.135 (0.033)	0.069 (0.045)

## Summary and conclusions

The overall objective of this study was to evaluate the effect of the TL supplemental nutritional labelling on the purchases of CSD by Ecuadorian consumers. We did not find evidence that the introduction of the TL labelling had reduced purchases of CSD in general or the overall purchases of high-sugar CSD in particular. We also did not find evidence of a change in consumers demand response to price changes due to the introduction of the TL label. We found a downward trend in purchases of high-sugar CSD and an upward trend in purchases of low- and non-sugar CSD. Additionally, households with higher socio-economic status tend to purchase less high-sugar and more low- and non-sugar soft drinks, suggesting they may have *healthier* food buying and consumption habits and that may also respond differently to the TL. Finally, we also find higher purchases of CSD during the first and second quarter of the year.

Some of the information generated in this study can be used to better target potential promotional efforts. First, the relationship between socio-economic status and high-sugar CSD purchases suggest that lower socio-economic status households should be the main target of any advertising campaigns promoting the use of the TL for healthy eating. Second, the observed seasonality effects, which show higher purchases of CSD during the first half of the year, suggest times of the year when promotional campaigns could be concentrated. It is well documented that the benefits and correct use of TL labels were initially promoted using a communication campaign through the media (radio, TV and other outlets) but information on the size and length of the campaign and its effectiveness is not available [42]. Any promotional efforts must consider the health literacy of the target group to efficiently promote the use of the TL. It is plausible that relegating the TL to the back panel of processed food products (instead of the front panel) and not targeted advertisement negatively affected the expected impact of the TL in the consumption of CSD, especially high-sugar CSD.

An alternative mechanism to promote changes in the buying and consumption habits of the population towards healthier food alternatives is the use of fiscal policies. Our estimated own-price elasticities indicate that Ecuadorians are sensitive to price changes, suggesting that a price increase as result of a tax on sugary beverages may reduce purchases consumption of high-sugar CSD. Moreover, a tax policy is unlikely to have the same effect across all CSD categories since the own-price elasticities of Coca-Cola are inelastic and the own-price elasticities of other high-sugar CSD and low- and non-sugar CSD are elastic.

Our study presents data limitations. We only have observations for 36 months, which disaggregated by income group yields a total of 108 observations that limited the number of degrees of freedom available to explore, for example, how socio-economic status affects the response to TL labelling. Future research could use household level panel data to explore the heterogeneity of effects of the TL label. Our results also only account for CSD purchases for consumption at home, which is only a portion of total purchases and consumption. Thus, more research is needed to evaluate the relationship between socio-economic status and the response to the TL labelling and to evaluate the effect of the TL label on soft drinks purchases and consumption away from home. Additionally, more research is also needed to evaluate the use of labels by consumers, the effect of TL labeling in other processed food products, and producers’ strategic responses to the policy (i.e., reformulation of products, marketing, and pricing). In spite of these limitations, relative to previous studies evaluating the effect of TL labels, this study has used a larger period of time as well as a theoretically consistent econometric framework, which allowed us to “control” for other factors affecting consumer demand for CSD. The presented framework may prove useful for future studies evaluating the impact of the introduction of supplementary nutritional labels.

To summarize our findings, we do not find evidence that the TL supplemental nutritional label for package foods implemented in Ecuador affected households’ CSD buying habits. This result is consistent with several experimental studies but, to the best of our knowledge, this is the first study using a nationally representative of actual consumer purchases.

## Supporting information

S1 Fig Supplemental nutritional information labels. (TIFF)

S2 Fig Shifts in the demand for carbonated soft drinks. (TIFF)

S3 Fig Carbonated soft drinks purchases. (TIFF)

S1 Table Nutrient content and traffic light color. (DOCX)

S2 Table Brands per category and average expenditures and quantities purchased before and after introduction of the policy. (DOCX)

S3 Table Descriptive statistics of carbonated soft drinks monthly quantity purchased and prices (January 2013 –December 2015). (DOCX)

S4 Table Marshallian price and expenditure elasticities. (DOCX)

S5 Table Effects of the demand shifters on mean expenditures ($ per-capita per month). (DOCX)

S6 Table Effects of demand shifters on mean quantities (L per-capita per month). (DOCX)

S7 Table Estimated effect of the introduction of the TL label on the demand for high sugar CSD (L/month per-capita). (DOCX)

S1 File TTU-Kantar research agreement. (PDF)

S1 Appendix Estimation of the demand system. (DOCX)

S2 Appendix Non-linear SUR parameter estimates. (DOCX)
