# Supplementary figures and images for: The effect of ‘Traffic-Light’ nutritional labelling in carbonated soft drink purchases in Ecuador
Source: PLoS One. 2019 Oct 3;14(10):e0222866. doi: 10.1371/journal.pone.0222866 (PMC6776320; doi:10.1371/journal.pone.0222866)

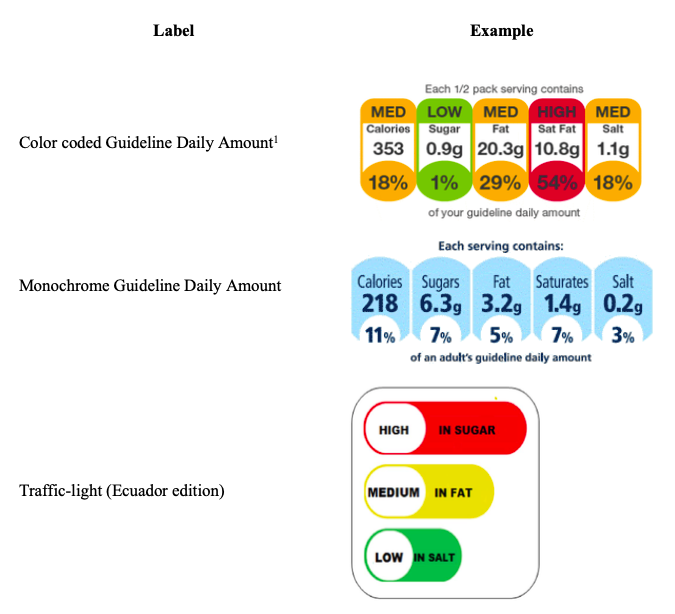

Supplement: S1 Fig — (TIFF) [file pone.0222866.s001.tiff]

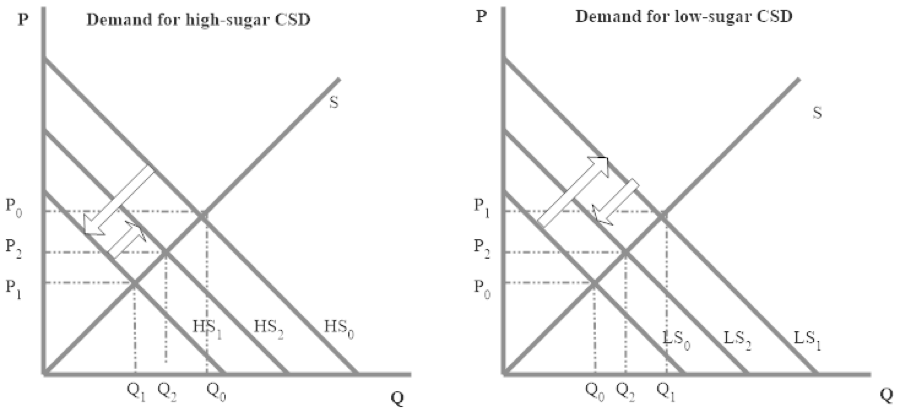

Supplement: S2 Fig — (TIFF) [file pone.0222866.s002.tiff]

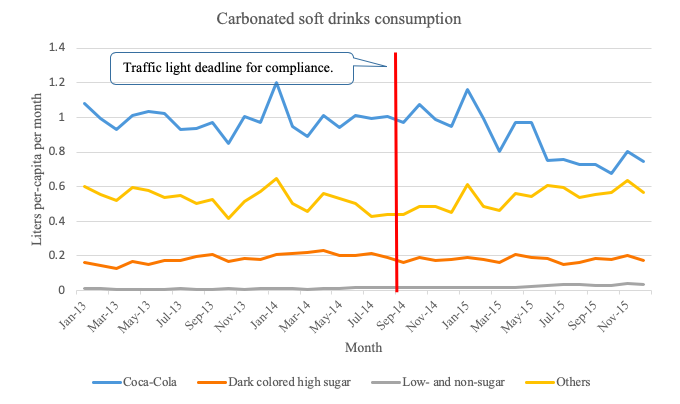

Supplement: S3 Fig — (TIFF) [file pone.0222866.s003.tiff]
