# Supplementary material for: The effect of ‘Traffic-Light’ nutritional labelling in carbonated soft drink purchases in Ecuador
Source: PLoS One. 2019 Oct 3;14(10):e0222866. doi: 10.1371/journal.pone.0222866 (PMC6776320; doi:10.1371/journal.pone.0222866)
Supplement: S1 Table — (DOCX) [file pone.0222866.s004.docx]

**Table 1. Nutrient content and traffic light color.**

| Component | Level | | |
| --- | --- | --- | --- |
|  | ‘Low’ concentration (Green) | ‘Medium’ concentration (Yellow) | ‘High’ concentration (Red) |
| Total fat | ≤ 3gr/100gr or ≤ 1.5gr/100ml | between 3 and 20gr/100gr or between 1.5 and 10 gr/100ml | ≥ 20gr/100gr or ≥ 10gr/100ml |
| Sugars | ≤ 5gr/100gr or ≤ 2.5gr/100ml | between 5 and 15gr/100gr or between 2.5 and 7.5 gr/100ml | ≥ 15gr/100gr or ≥ 7.5gr/100ml |
| Salt (Sodium) | ≤ 120 mg/100gr or ≤ 120 mg/100ml | between 120 and 600mg/100gr or between 120 and 600 mg/100ml | ≥ 600mg/100gr or ≥ 600 mg/100ml |

Source: Ecuadorian technical regulation RTE INEN 022 (2R) [3]
