# Supplementary material for: The effect of ‘Traffic-Light’ nutritional labelling in carbonated soft drink purchases in Ecuador
Source: PLoS One. 2019 Oct 3;14(10):e0222866. doi: 10.1371/journal.pone.0222866 (PMC6776320; doi:10.1371/journal.pone.0222866)
Supplement: S2 Table — (DOCX) [file pone.0222866.s005.docx]

**Table 2. Brands per category and average expenditures and quantities purchased before and after introduction of the policy.**

|  | Mean per-capita monthly expenditures (U.S.$)* | | Mean per-capita monthly quantity purchased (L) | |  |
| --- | --- | --- | --- | --- | --- |
| Category | Before | After | Before | After | Brands/types of product |
| Coca-Cola | 0.67 | 0.58 | 0.99 | 0.88 | Coca-Cola |
| Dark colored high sugar | 0.07 | 0.08 | 0.19 | 0.18 | Pepsi and Big-Cola |
| Low- and non-sugar | 0.04 | 0.04 | 0.01 | 0.03 | Coca-Cola light, Coca-Cola zero, Sprite zero, Inca-Kola and Barrilitos-O-Key. |
| All other high sugar sodas | 0.31 | 0.28 | 0.53 | 0.54 | Coca-Cola life, Fanta, Frioravanti, 7up, Mas, Kola gallito, Oro, Tropical, Quintuples, Orangine, Fox Cola, Fruit and all other. |
| Total | 1.09 | 0.98 | 1.72 | 1.63 | All |

*The period before corresponds from January 2013 to August 2014 (on August 29th all medium and large companies were required to comply with the TL labelling) and the period after corresponds from September 2014 to December 2015.
