# Supplementary material for: The effect of ‘Traffic-Light’ nutritional labelling in carbonated soft drink purchases in Ecuador
Source: PLoS One. 2019 Oct 3;14(10):e0222866. doi: 10.1371/journal.pone.0222866 (PMC6776320; doi:10.1371/journal.pone.0222866)
Supplement: S3 Table — (DOCX) [file pone.0222866.s006.docx]

**Table 3. Descriptive statistics of carbonated soft drinks monthly quantity purchased and prices (January 2013- December 2015).**

| Brand |  | Volume - Liters per-capita | | | | Price - US$ per-liter | | | |
| --- | --- | --- | --- | --- | --- | --- | --- | --- | --- |
|  | N | Mean | Std. Dev. | Min. | Max. | Mean | Std. Dev. | Min. | Max. |
| Coca-Cola | 108 | 0.958 | 0.150 | 0.639 | 1.344 | 0.651 | 0.033 | 0.496 | 0.769 |
| Coca-Cola Life | 108 | 0.001 | 0.003 | 0.000 | 0.015 | 0.868 | 0.108 | 0.696 | 1.110 |
| Coca-Cola Zero | 108 | 0.008 | 0.008 | 0.000 | 0.028 | 0.695 | 0.099 | 0.134 | 0.925 |
| Coca-Cola Light | 108 | 0.017 | 0.012 | 0.000 | 0.053 | 0.759 | 0.090 | 0.576 | 1.400 |
| Fanta | 108 | 0.055 | 0.013 | 0.029 | 0.095 | 0.663 | 0.036 | 0.591 | 0.841 |
| Fioravanti | 108 | 0.106 | 0.025 | 0.054 | 0.155 | 0.635 | 0.029 | 0.572 | 0.707 |
| Sprite | 108 | 0.136 | 0.031 | 0.077 | 0.217 | 0.660 | 0.026 | 0.609 | 0.834 |
| Sprite Zero | 108 | 0.006 | 0.06 | 0.000 | 0.024 | 0.689 | 0.126 | 0.097 | 1.235 |
| Inca Kola Regular | 108 | 0.026 | 0.018 | 0.003 | 0.102 | 0.623 | 0.055 | 0.427 | 0.757 |
| Pepsi | 108 | 0.065 | 0.023 | 0.023 | 0.112 | 0.562 | 0.035 | 0.515 | 0.732 |
| 7Up | 108 | 0.022 | 0.008 | 0.006 | 0.047 | 0.577 | 0.048 | 0.487 | 0.771 |
| Mas | 108 | 0.018 | 0.007 | 0.006 | 0.035 | 0.543 | 0.045 | 0.391 | 0.764 |
| Kola Gallito | 108 | 0.016 | 0.009 | 0.003 | 0.046 | 0.532 | 0.060 | 0.391 | 1.005 |
| Big Cola | 108 | 0.071 | 0.061 | 0.002 | 0.202 | 0.531 | 0.054 | 0.443 | 0.833 |
| Oro | 108 | 0.006 | 0.010 | 0.000 | 0.037 | 0.525 | 0.065 | 0.432 | 0.833 |
| Tropical | 108 | 0.059 | 0.015 | 0.032 | 0.099 | 0.572 | 0.029 | 0.500 | 0.650 |
| Manzana | 108 | 0.042 | 0.012 | 0.017 | 0.076 | 0.562 | 0.034 | 0.437 | 0.642 |
| Quintuples | 108 | 0.012 | 0.005 | 0.002 | 0.035 | 0.599 | 0.065 | 0.453 | 0.853 |
| Oranguine | 108 | 0.005 | 0.003 | 0.000 | 0.024 | 0.499 | 0.069 | 0.333 | 0.756 |
| Fox Cola | 108 | 0.002 | 0.002 | 0.000 | 0.008 | 0.504 | 0.150 | 0.188 | 1.369 |
| Barrilitos-O-Key | 108 | 0.002 | 0.003 | 0.000 | 0.012 | 0.484 | 0.175 | 0.267 | 1.126 |
| Fruit | 108 | 0.001 | 0.001 | 0.000 | 0.005 | 0.529 | 0.156 | 0.272 | 1.111 |
| Others | 108 | 0.002 | 0.002 | 0.000 | 0.010 | 0.628 | 0.250 | 0.075 | 1.747 |
