# Supplementary material for: The effect of ‘Traffic-Light’ nutritional labelling in carbonated soft drink purchases in Ecuador
Source: PLoS One. 2019 Oct 3;14(10):e0222866. doi: 10.1371/journal.pone.0222866 (PMC6776320; doi:10.1371/journal.pone.0222866)
Supplement: S4 Table — (DOCX) [file pone.0222866.s007.docx]

**Table 4. Marshallian price and expenditure elasticities.**

|  | Coca-Cola | Dark colored high-sugar | Low- and non-sugar | All other high sugar sodas | All other foods | Expenditure elasticities |
| --- | --- | --- | --- | --- | --- | --- |
| Coca-Cola | -0.597 | -0.315 | 0.258 | 0.447 | -0.435 | 0.641 |
|  | (0.487) | (0.081) | (0.090) | (0.256) | (0.239) | (0.115) |
| Dark colored high-sugar | -2.464 | -1.955 | 0.417 | 3.463 | -0.252 | 0.791 |
|  | (0.627) | (0.449) | (0.292) | (0.797) | (0.570) | (0.210) |
| Low- and non-sugar | 4.369 | 0.905 | -2.806 | -3.211 | 0.755 | -0.012 |
|  | (1.569) | (0.651) | (0.643) | (1.372) | (1.213) | (0.420) |
| All other high sugar sodas | 0.940 | 0.933 | -0.402 | -1.244 | -0.767 | 0.540 |
|  | (0.542) | (0.214) | (0.167) | (0.627) | (0.365) | (0.148) |
| All other foods | -0.021 | -0.002 | 0.000 | -0.015 | -0.979 | 1.018 |
|  | (0.007) | (0.002) | (0.002) | (0.005) | (0.009) | (0.004) |

Standard errors in parenthesis.
