# Supplementary material for: The effect of ‘Traffic-Light’ nutritional labelling in carbonated soft drink purchases in Ecuador
Source: PLoS One. 2019 Oct 3;14(10):e0222866. doi: 10.1371/journal.pone.0222866 (PMC6776320; doi:10.1371/journal.pone.0222866)
Supplement: S5 Table — (DOCX) [file pone.0222866.s008.docx]

**Table 5. Effects of the demand shifters on mean expenditures ($ per-capita per month).**

|  | High socio-economic status | Medium socio-economic status | Time trend | **Traffic light labelling** | 1^st^ quarter | 2^nd^ quarter | 3^rd^ quarter |
| --- | --- | --- | --- | --- | --- | --- | --- |
| Coca-Cola | -0.088 | 0.015 | -0.007 | 0.090 | 0.072 | 0.035 | -0.019 |
|  | (0.032) | (0.025) | (0.001) | (0.019) | (0.016) | (0.014) | (0.014) |
| Dark colored high-sugar | -0.117 | -0.082 | 0.001 | -0.015 | 0.004 | 0.003 | -0.003 |
|  | (0.008) | (0.006) | (0.000) | (0.006) | (0.004) | (0.004) | (0.003) |
| Low- and non-sugar | 0.023 | 0.009 | 0.002 | -0.005 | -0.004 | -0.001 | -0.002 |
|  | (0.007) | (0.006) | (0.000) | (0.005) | (0.003) | (0.003) | (0.004) |
| All other high sugar sodas | -0.097 | -0.013 | 0.000 | 0.010 | 0.031 | 0.022 | -0.017 |
|  | (0.020) | (0.016) | (0.001) | (0.014) | (0.012) | (0.009) | (0.009) |

Standard errors in parenthesis.
Note: Rows in the table include marginal effects corresponding to each of the four demand equations estimated in the system. The baseline category for socio-economic status effects is the low socio-economic status. Baseline category for the effects of quarters is the 4^th^ quarter.
