# Supplementary material for: The effect of ‘Traffic-Light’ nutritional labelling in carbonated soft drink purchases in Ecuador
Source: PLoS One. 2019 Oct 3;14(10):e0222866. doi: 10.1371/journal.pone.0222866 (PMC6776320; doi:10.1371/journal.pone.0222866)
Supplement: S6 Table — (DOCX) [file pone.0222866.s009.docx]

**Table 6. Effects of demand shifters on mean quantities (L per-capita per month).**

|  | High socio-economic status | Medium socio-economic status | Time trend | **Traffic light labelling** | 1^st^ quarter | 2^nd^ quarter | 3^rd^ quarter |
| --- | --- | --- | --- | --- | --- | --- | --- |
| Coca-Cola | -0.132 | 0.022 | -0.011 | 0.135 | 0.109 | 0.053 | -0.029 |
|  | (0.048) | (0.038) | (0.002) | (0.028) | (0.025) | (0.021) | (0.021) |
| Dark colored high-sugar | -0.193 | -0.135 | 0.001 | -0.025 | 0.006 | 0.004 | -0.004 |
|  | (0.013) | (0.010) | (0.000) | (0.009) | (0.006) | (0.006) | (0.005) |
| Low- and non-sugar | 0.035 | 0.013 | 0.002 | -0.008 | -0.006 | -0.001 | -0.004 |
|  | (0.011) | (0.009) | (0.000) | (0.007) | (0.005) | (0.005) | (0.005) |
| All other high sugar sodas | -0.151 | -0.020 | 0.000 | 0.016 | 0.048 | 0.035 | -0.026 |
|  | (0.031) | (0.025) | (0.001) | (0.022) | (0.018) | (0.014) | (0.014) |

Standard errors in parenthesis.
Note: Rows in the table include marginal effects corresponding to each of the four demand equations estimated in the system. The baseline category for socio-economic status effects is the low socio-economic status. Baseline category for the effects of quarters is the 4^th^ quarter.
