# Supplementary material for: The effect of ‘Traffic-Light’ nutritional labelling in carbonated soft drink purchases in Ecuador
Source: PLoS One. 2019 Oct 3;14(10):e0222866. doi: 10.1371/journal.pone.0222866 (PMC6776320; doi:10.1371/journal.pone.0222866)
Supplement: S7 Table — (DOCX) [file pone.0222866.s010.docx]

**Table 7. Estimated effect of the introduction of the TL label on the demand for high sugar CSD (L/month per-capita).**

|  | Potential break points in the demand for CSD | | |
| --- | --- | --- | --- |
| Model | 18 | 20 | 24 |
| AIDS | 0.084 (0.040) | 0.127 (0.037) | 0.058 (0.036) |
| Rotterdam | 0.006 (0.052) | 0.009 (0.054) | 0.003 (0.105) |
| EASI | 0.096 (0.043) | 0.135 (0.033) | 0.069 (0.045) |
