# Supplementary material for: The effect of ‘Traffic-Light’ nutritional labelling in carbonated soft drink purchases in Ecuador
Source: PLoS One. 2019 Oct 3;14(10):e0222866. doi: 10.1371/journal.pone.0222866 (PMC6776320; doi:10.1371/journal.pone.0222866)
Supplement: S1 File — (PDF) [file pone.0222866.s011.pdf]

KANTAR WORLDPANEL

## Texas Tech University (TTU) Third Party Research Agreement

| | | | |
|---|---|---|---|
| **TTU Collaborator:** | Carlos E. Carpio | **Project Name:** | Impact of 'traffic-light' nutrition labeling on consumer food purchases in Ecuador. |
| **Address:** | 2500 Broadway, Lubbock, TX 79409, United States | **Project Start Date:** | April 15, 2016 |
| **Phone:** | +1 806-742-2011 | **Expected Project End Date:** | August 15, 2017 |
| **TTU Researchers:** | Department of Agricultural and Applied Economics, Texas Tech University | **Licensed Materials:** | Consumer panel data, data dictionaries and data methodologies. |

### TERMS AND CONDITIONS

**Ownership License.** All Kantar Worldpanel Licensed Materials provided by Texas Tech University ("TTU") and shared with its research collaborator listed above ("Collaborator") are owned by Kantar Worldpanel. Licensed Materials include reports, data, analyses ("Information"); data dictionaries, reference tools, data methodologies, disaggregated data, data characteristics and attributes ("References"); software ("Software") or other intellectual property ("Property"), collectively, the "Licensed Materials." Collaborator holds a limited, non-exclusive license to make use of the Licensed Materials solely for the specific academic research purpose for which Collaborator has been retained by TTU. Collaborator may include excerpts of Information in its research results for TTU and/or for disclosure in academic journals and TTU publications. Collaborator will use Kantar Worldpanel's Licensed Materials ONLY for specified research on behalf of TTU. Collaborator may not use the Licensed Materials for research outside of the topics and issues that have been pre-specified. Collaborator agrees to indemnify and hold Kantar Worldpanel harmless as stated below and to make available to Kantar Worldpanel the results of Collaborator's published research. Subject to copyrights, Kantar Worldpanel may use and disclose the published results in its business. In all published writings by Collaborator, any portions of the Licensed Materials that are disclosed must show Kantar Worldpanel's copyright; be accurately labeled; and not be presented in a misleading manner. Notwithstanding anything to the contrary, Retailer-specific Information may not be disclosed by Collaborator.

**Prohibition Against Non-Specified Use.** Any use or disclosure other than as specified in this license is prohibited without Kantar Worldpanel's prior written consent.

**Charges.** TTU is responsible for only those charges associated with its license grant, pursuant to a separate agreement with Kantar Worldpanel. No additional charges will apply pursuant to this Agreement; provided that Kantar Worldpanel may suspend this license in the event of nonpayment by TTU under its agreement with Kantar Worldpanel.

**Indemnification.** Because Kantar Worldpanel products and services are intended for internal use by Collaborator, Collaborator agrees to indemnify and hold harmless Kantar Worldpanel, its officers and directors against all claims, damages, loss or expenses arising from Collaborator's disclosure of Information or from Collaborator's use of the Licensed Materials other than in strict compliance with the terms and conditions of this Agreement.

**Accuracy.** Information consists primarily of estimates, representing Kantar Worldpanel's opinion, based on projections using statistical procedures Kantar Worldpanel deems appropriate. It is subject to inherent coverage, error rate and other statistical factors. Sources of information are not always under Kantar Worldpanel's control.

**Limitation of Liability.** Because this license is granted by Kantar Worldpanel as an accommodation to TTU, Collaborator agrees that Kantar Worldpanel will not be liable, whether in contract, tort (including negligence) or otherwise, for any loss, expense, injury or damage of any kind including, without limitation, any actual, direct, indirect, special, incidental or consequential damages, whether suffered by Collaborator, or any other person or entity, even if Kantar Worldpanel is advised or has knowledge of the possibility thereof, resulting, directly or indirectly, from Kantar Worldpanel's failure to furnish any Licensed Materials; any errors, inaccuracies or omissions in the Licensed Materials; or the use of the Licensed Materials by Collaborator or others. Collaborator agrees that Collaborator's sole and exclusive remedy will be the correction of erroneous Information and References where feasible if timely brought to Kantar Worldpanel's attention. These are Collaborator's exclusive remedies.

**Termination.** The license granted to Collaborator under this Agreement remains in effect until the soonest of (a) TTU no longer has a contractual right to obtain Licensed Materials from Kantar Worldpanel; (b) TTU has terminated its agreement with Collaborator; or (c) the research project performed by Collaborator for TTU has ended. Kantar Worldpanel may terminate this Agreement at any time upon written notice to Collaborator. Upon termination, Collaborator shall return, destroy or purge all copies of the Licensed Materials and provide written certification of destruction and purging.

**General Provisions.** This Agreement and the parties' respective rights and duties shall be interpreted and governed in accordance with the laws of the State of Illinois.

| | | | |
|---|---|---|---|
| AGREED TO ON BEHALF OF COLLABORATOR | **BY: Carlos E. Carpio** | **DATE:** | April 11th, 2016 |
| AGREED TO ON BEHALF OF KANTAR WORLDPANEL | **BY: Mario Arregui** | **DATE:** | **April 7th, 2016** |

**Kantar Worldpanel – Kantar Worldpanel Ecuador Kantarecsa S.A**
**Av. Naciones Unidas E9-74 entre Av. Shyris y República del Salvador. Edificio Ordoñez Robayo, Penthouse.**
**Quito, EC 170505**
