# Supplementary material for: The effect of ‘Traffic-Light’ nutritional labelling in carbonated soft drink purchases in Ecuador
Source: PLoS One. 2019 Oct 3;14(10):e0222866. doi: 10.1371/journal.pone.0222866 (PMC6776320; doi:10.1371/journal.pone.0222866)
Supplement: S1 Appendix — (DOCX) [file pone.0222866.s012.docx]

**Appendix 1. Estimation of the demand system.**

The AIDS demand system specification is

$w_{iht}=\alpha_{ih}+\sum_{j=1}^{5} \gamma_{ij}\ln p_{jht}+\beta_{i}\ln\left( \frac{E_{ht}}{P_{ht}} \right)+\boldsymbol{\delta}_{\mathbf{ih}}\mathbf{'}\mathbf{z}_{\mathbf{iht}}+\varepsilon_{ikt}$,

where *i* the index for soft drink groups, *h* is the index for household socio-economic status group, and *t* is the time period. $w_{iht}$ are the budget shares, $p_{jht}$ are soft drink prices, $E_{ht}$ is total per-capita monthly expenditures in food and drinks, $P_{ht}$ is a price index, $\mathbf{z}_{\mathbf{iht}}$is a vector of other factors affecting demand including a time trend, socio-economic status of the household, quarterly dummy variables, and a dummy variable for the introduction of the TL labelling policy. The $\alpha_{i}'s, \gamma_{ij}'s$, $\beta_{i}'s$, and $\boldsymbol{\delta}_{\mathbf{i}}\boldsymbol{'s}$ are model parameters.

The price index is of the form:

$$\ln P_{ht}=\sum_{j=1}^{5} \alpha_{j}\ln p_{jht}+\frac{1}{2}\sum_{j=1}^{5} \sum_{i=1}^{5} \gamma_{ij}\ln p_{iht}\ln p_{jht}$$

Our demand system consists of 5 equations, 4 corresponding to different CSD groups and the 5^th^ one to a numeraire good that includes all other foods and beverages consumed by the households in the panel. Prices used in the demand systems are Fisher price indices, $p_{j}=\sqrt{\frac{\sum p_{kt}q_{k0}}{\sum p_{k0}q_{k0}}\frac{\sum p_{kt}q_{kt}}{\sum p_{k0}q_{kt}}}$, for brands/food type *k =* 1 to K and period *t =* 1 to 36, where *p_kt_* is price food *k* in period *t* and *q_kt_* is quantities of food *k* in period *t*.

Although a larger demand system could be considered, the lack of degrees of freedom precludes the estimation of such a system.

Uncompensated price elasticities were estimated using the following equation:

1. $e_{ij}=\psi_{ij}+\frac{\gamma_{ij}}{w_{i}}-\beta_{i}\frac{\alpha_{j}}{w_{i}}-\frac{\beta_{i}}{w_{i}}\sum_{j=1}^{5} \gamma_{ij}\ln p_{j}$

where $\psi_{ij}$ is the Kronecker delta ($\psi_{ij}=-1$ for *i = j* and $\psi_{ij}=0$ for *i ≠ j*).

The expenditure elasticity was estimated using the formula

1. $\eta_{i}=1+\frac{\beta_{i}}{w_{i}}$.

To evaluate the effect of factors other than prices and total expenditures on expenditures and quantity demanded (i.e., the shift effect in the demand curve) of the *i*th good, we use the following formulas (derivation shown below):

1. ${\Delta Expenditures}_{ik}=\delta_{ik}E$,
2. ${\Delta Quantities}_{ik}=\frac{\delta_{ik}E}{p_{i}}$,

where the *ik* sub-index denotes the effect of the *kth* factor on vector $\mathbf{z}_{\mathbf{ik}}$ on the demand for the *ith* good.

The effect of the TL label or any other factor on $\mathbf{z}_{\mathbf{ik}}$ on the slope of the demand curve for the *i*th good can be evaluated using the derivative of the own price elasticity, $e_{ii},$with respect to the factor of interest, $z_{ik}$ [24]:

$$7) \frac{\partial e_{ii}}{{\partial z}_{ik}}=-\frac{\left( \gamma_{ij}- \beta_{i}\alpha_{j}-\beta_{i}\sum_{j=1}^{5} \gamma_{ij}\ln p_{j} \right)}{w_{i}^{2}}\delta_{ik}.$$

Effect on expenditures for category i

Let $\delta_{ik}=\frac{\partial w_{i}}{\partial z_{ik}}$, where $w_{i}=\frac{E_{i}}{E}$,

Therefore $\frac{\partial w_{i}}{\partial z_{ik}}=\frac{\frac{1}{E}\partial E_{i}}{\partial z_{ik}}$,

Re-arranging the terms we get: $\frac{\partial w_{i}}{\partial z_{ij}}=\frac{\partial E_{i}}{\partial z_{ij}}\frac{1}{E}$,

And because $\delta_{ij}=\frac{\partial w_{i}}{\partial z_{ij}}$, we have that $\delta_{ij}E=\frac{\partial E_{i}}{\partial z_{ij}}$

Effect on quantities for category i

Let $\delta_{ij}=\frac{\partial w_{i}}{\partial z_{ij}}$, where $w_{i}=\frac{q_{i}p_{i}}{E}$,

therefore $\frac{\partial w_{i}}{\partial z_{ij}}=\frac{\frac{p_{i}}{E}\partial q_{i}}{\partial z_{ij}}$,

Re-arranging the terms we get: $\frac{\partial w_{i}}{\partial z_{ij}}=\frac{\partial q_{i}}{\partial z_{ij}}\frac{p_{i}}{E}$,

And because $\delta_{ij}=\frac{\partial w_{i}}{\partial z_{ij}}$, we have that $\frac{\delta_{ij}E}{p_{i}}=\frac{\partial q_{i}}{\partial z_{ij}}$
