# Supplementary material for: The effect of ‘Traffic-Light’ nutritional labelling in carbonated soft drink purchases in Ecuador
Source: PLoS One. 2019 Oct 3;14(10):e0222866. doi: 10.1371/journal.pone.0222866 (PMC6776320; doi:10.1371/journal.pone.0222866)
Supplement: S2 Appendix — (DOCX) [file pone.0222866.s013.docx]

**Appendix 2. Non-linear SUR parameter estimates.**

| Description | Variable | B | SE B | p |
| --- | --- | --- | --- | --- |
| Prices | $\gamma_{11}$ | 0.0099 | 0.0115 | 0.3859 |
|  | $\gamma_{12}$ | -0.0080 | 0.0019 | 0.0000 |
|  | $\gamma_{13}$ | 0.0065 | 0.0023 | 0.0040 |
|  | $\gamma_{14}$ | 0.0112 | 0.0060 | 0.0611 |
|  | $\gamma_{15}$ | -0.0197 | 0.0061 | 0.0013 |
|  | $\gamma_{22}$ | -0.0031 | 0.0014 | 0.0308 |
|  | $\gamma_{23}$ | 0.0014 | 0.0010 | 0.1746 |
|  | $\gamma_{24}$ | 0.0112 | 0.0029 | 0.0001 |
|  | $\gamma_{25}$ | -0.0015 | 0.0023 | 0.5245 |
|  | $\gamma_{33}$ | -0.0027 | 0.0010 | 0.0065 |
|  | $\gamma_{34}$ | -0.0049 | 0.0020 | 0.0140 |
|  | $\gamma_{35}$ | -0.0003 | 0.0019 | 0.8683 |
|  | $\gamma_{44}$ | -0.0030 | 0.0073 | 0.6794 |
|  | $\gamma_{45}$ | -0.0146 | 0.0050 | 0.0036 |
|  | $\gamma_{55}$ | 0.0360 | 0.0098 | 0.0002 |
| Intercepts | $\alpha_{1}$ | 0.0288 | 0.0012 | 0.0000 |
|  | $\alpha_{2}$ | 0.0056 | 0.0003 | 0.0000 |
|  | $\alpha_{3}$ | 0.0001 | 0.0002 | 0.7708 |
|  | $\alpha_{4}$ | 0.0129 | 0.0006 | 0.0000 |
|  | $\alpha_{5}$ | 0.9526 | 0.0014 | 0.0000 |
| Expenditures | $\beta_{1}$ | -0.0091 | 0.0029 | 0.0017 |
|  | $\beta_{2}$ | -0.0007 | 0.0007 | 0.3375 |
|  | $\beta_{3}$ | -0.0015 | 0.0006 | 0.0155 |
|  | $\beta_{4}$ | -0.0056 | 0.0018 | 0.0024 |

Appendix 2. Continued

| Description | Variable | B | SE B | p |
| --- | --- | --- | --- | --- |
| High socio-economic status | $\delta_{11}$ | -0.0035 | 0.0013 | 0.0068 |
|  | $\delta_{21}$ | -0.0046 | 0.0003 | 0.0000 |
|  | $\delta_{31}$ | 0.0009 | 0.0003 | 0.0016 |
|  | $\delta_{41}$ | -0.0038 | 0.0008 | 0.0000 |
| Medium socio-economic status | $\delta_{12}$ | 0.0006 | 0.0010 | 0.5611 |
|  | $\delta_{22}$ | -0.0032 | 0.0002 | 0.0000 |
|  | $\delta_{32}$ | 0.0003 | 0.0002 | 0.1284 |
|  | $\delta_{42}$ | -0.0005 | 0.0006 | 0.4024 |
| Time trend | $\delta_{13}$ | -0.0003 | 0.0000 | 0.0000 |
|  | $\delta_{23}$ | 0.0000 | 0.0000 | 0.0077 |
|  | $\delta_{33}$ | 0.0001 | 0.0000 | 0.0000 |
|  | $\delta_{43}$ | 0.0000 | 0.0000 | 0.9738 |
| Traffic-light labelling | $\delta_{14}$ | 0.0035 | 0.0007 | 0.0000 |
|  | $\delta_{24}$ | -0.0006 | 0.0002 | 0.0178 |
|  | $\delta_{34}$ | -0.0002 | 0.0002 | 0.2589 |
|  | $\delta_{44}$ | 0.0004 | 0.0006 | 0.4868 |
| Seasonal | $\delta_{15}$ | 0.0014 | 0.0006 | 0.0175 |
|  | $\delta_{25}$ | 0.0001 | 0.0002 | 0.3609 |
|  | $\delta_{35}$ | -0.0002 | 0.0001 | 0.1566 |
|  | $\delta_{45}$ | 0.0012 | 0.0005 | 0.0073 |
|  | $\delta_{16}$ | 0.0014 | 0.0006 | 0.0175 |
|  | $\delta_{26}$ | 0.0001 | 0.0002 | 0.5036 |
|  | $\delta_{36}$ | 0.0000 | 0.0001 | 0.8175 |
|  | $\delta_{46}$ | 0.0009 | 0.0003 | 0.0081 |
|  | $\delta_{17}$ | -0.0008 | 0.0005 | 0.1601 |
|  | $\delta_{27}$ | -0.0001 | 0.0001 | 0.4237 |
|  | $\delta_{37}$ | -0.0001 | 0.0001 | 0.4820 |
|  | $\delta_{47}$ | -0.0007 | 0.0003 | 0.0509 |
